# Homologous recombination and evolutionary arms race drive the adaptive evolution of African swine fever virus

**DOI:** 10.1186/s13567-025-01573-1

**Published:** 2025-07-08

**Authors:** Wenqiang Wang, Qinqiu Liu, Qilin Zhao, Zhenbang Zhu, Wei Wen, Zhendong Zhang, Xiangdong Li

**Affiliations:** 1 Jiangsu Co-innovation Center for Prevention and Control of Important Animal Infectious Diseases and Zoonoses, College of Veterinary Medicine, Yangzhou University, Yangzhou, China; 2 Joint International Research Laboratory of Agriculture and Agri-Product Safety, The Ministry of Education of China, Yangzhou University, Yangzhou, China

**Keywords:** ASFV, evolution, core and non-core genes, homologous recombination, evolutionary arms race

## Abstract

African Swine Fever Virus (ASFV) is a highly contagious pathogen responsible for substantial economic losses in swine populations worldwide. Despite extensive research, the mechanisms underlying the genomic evolution of ASFV remain poorly understood. In this study, we conducted a comprehensive analysis of ASFV evolutionary strategies by examining 252 complete ASFV genomes. Our pan-genome analysis categorizes ASFV genes into core and non-core categories, with core genes predominantly locate in the central region of the genome, while non-core genes are primarily situated at the variable genomic termini, exhibiting higher rates of genetic loss and diversification. Gene synteny analysis revealed that ASFV inherited a portion of its core gene repertoire from the common ancestor of the *Asfarviridae* family, establishing its central genomic framework, and acquired virus-specific genes that contributed to its distinct genetic identity during divergence. Homologous recombination analysis identified 76 genes exhibiting strong recombination signals, emphasizing the critical role of recombination in ASFV evolution. Additionally, 9 genes were found to be under positive selection, highlighting the influence of the host-virus evolutionary arms race in shaping ASFV genome, particularly in terms of immune evasion and host interaction. These findings underscore the dynamic evolutionary forces driving ASFV adaptive evolution and provide important implications for understanding the virus global spread and the development of effective control measures.

**Supplementary Information:**

The online version contains supplementary material available at 10.1186/s13567-025-01573-1.

## Introduction

African Swine Fever Virus (ASFV) is a highly contagious and economically devastating pathogen that causes African Swine Fever (ASF), a disease characterized by high mortality rates in swine populations [1, 2]. ASFV poses a major threat to global pig farming, with outbreaks resulting in massive economic losses and trade restrictions [3]. The ongoing spread and persistence of ASFV across various regions have drawn attention to its remarkable ability to adapt to different environmental and host conditions.

ASFV belongs to the *Asfarviridae* family, which is part of the broader group of Nucleocytoplasmic Large DNA Viruses (NCLDVs) [4]. The ASFV genome is a large, double-stranded DNA molecule ranging from 170 to 193 kb in length, containing between 151 and 186 open reading frames (ORFs) [5, 6]. The genome is structured into three main regions: a left variable region (LVR), a conserved central region (CCR), and a right variable region (RVR) [7, 8], which together contribute to the genetic diversity of the virus and its adaptability to different environments. Understanding the genetic structure and evolutionary mechanisms of ASFV is critical for developing effective strategies to combat the disease, especially in light of the challenges posed by its persistence and rapid spread. 

Despite decades of research, the mechanisms driving the genomic evolution of ASFV remain poorly understood. Much of the current research has focused on the virus molecular epidemiology, phylogeny, and the identification of virulence factors [9–11]. Phylogenetic studies have revealed that ASFV strains fall into distinct genotypes with varying geographic distributions, highlighting the capacity of ASFV to diversify over time [12, 13]. While advances have been made in describing the diversity of ASFV strains, the underlying processes driving this variability remain elusive.

The evolutionary forces driving viral genome adaptation are diverse and multifaceted. One of the most significant mechanisms contributing to viral evolution is recombination, where genetic material from different strains or genotypes is exchanged, resulting in new viral variants [14–16]. This process allows viruses like ASFV to acquire advantageous traits, such as improved immune evasion capabilities or increased transmission efficiency. Another driving force is the evolutionary arms race between the virus and its host [17]. As hosts develop immune responses to combat viral infections, viruses must evolve rapidly to escape immune detection [18]. This constant selection pressure can drive the virus to evolve rapidly, equipping it with enhanced capabilities to survive in the host environment. However, like other double-stranded DNA viruses, ASFV usually exhibits a low degree of nucleotide substitution due to the proofreading mechanisms [19]. Hence, the evolutionary dynamics of ASFV under the influence of two opposing evolutionary forces remain poorly understood. To address these questions, a deeper investigation into ASFV genomic structure, dynamics of recombination events, and pathways that lead to adaptive variation is necessary.

In this study, we performed detailed analyses of the genetic architecture of ASFVs, exploring the evolutionary dynamics through the examination of complete genome sequences. By focusing on core genes, genomic synteny, homologous recombination, and selection pressures, we discovered that ASFVs possess a core genetic composition, part of which is inherited from a common ancestor of the *Asfarviridae* family. Additionally, homologous recombination and the evolutionary arms race play a prominent role in driving the evolution of ASFV. Our findings not only contribute to a better understanding of ASFV evolution but also have theoretical implications for the control and prevention of ASFV outbreaks.

## Materials and methods

### Phylogenetic analyses

All ASFV genome sequences were retrieved from the NCBI database, comprising a total of 252 complete genomes. The sequences were aligned using MAFFT v7.518 with the L-INS-I algorithm [20]. A phylogenetic tree was subsequently reconstructed using a maximum likelihood (ML) approach implemented in IQ-TREE version 2.1.2, with 1000 ultrafast bootstrap replicates [21]. The best-fit substitution model was selected using ModelFinder, integrated within IQ-TREE 2.1.2, based on the Bayesian Information Criterion (BIC). Considering that recombinant ASFV strains derived from genotypes I and II challenge the reliability of single-gene classification, we adopted a whole-genome-based phylogenetic approach to define the major lineages in this study.

### Identification of core genes and non-core genes

To interrogate the extent and patterns of gene variability across ASFV strains, we used the Genome Annotation Transfer Utility (GATU) software to annotate the ASFV genome [22], using a reference genome as a template. Three reference genomes were selected based on ASFV types: Type I (Reference: NC_044941.1), Type II (Reference: MK333180.1), and Recombinant (Reference: OQ504955.1). The reference genomes were downloaded from the GenBank database in GenBank format, containing gene annotations. Default parameters were applied during the annotation process to ensure standardization and consistency. The annotation results included information on the location of coding regions, functional predictions, and gene name assignments. Based on the annotation results, gene information was compiled and analyzed. In order to attenuate the impact of sequencing quality, genes present in more than 95% of ASFV strains were classified as core genes, while those present in fewer than 95% were categorized as non-core genes. Additionally, genes were classified according to ASFV genotyping into genotype I core and non-core genes, and genotype II core and non-core genes.

### Gene synteny analyses

We conducted gene synteny analyses across five members of the *Asfarviridae* family, including ASFV, ABALV, Pacmanvirus, Faustovirus, and Kaumoebavirus. Genomic sequences and annotations for these viruses were retrieved from NCBI database. To investigate the gene relationships within the *Asfarviridae* family, each gene in the ASFV genome was queried against the genomes of ABALV, Pacmanvirus, Faustovirus, and Kaumoebavirus using tBLASTN. For synteny analysis, the genomic collinearity among the five viruses was visualized using GenomeSyn [23].

### Homologous recombination analyses

To elucidate the role of recombination in ASFV evolution, we performed recombination analyses across all ASFV genes. We applied CD-HIT clustering to the complete set of 252 ASFV genomes using a 98% sequence identity threshold [24]. One representative genome was then selected from each resulting cluster for downstream recombination analysis. Subsequently, each ASFV gene, regardless of whether it was a core gene, was used as a query to perform BLAST searches against the representative ASFV genomes, obtaining the corresponding gene DNA sequences. Genes yielding fewer than 10 BLAST hits were excluded from further analysis. MAFFT v7.518 were used for multiple sequence alignment [20]. To identify potential homologous recombination events within the ASFV genomes, RDP4 software was utilized to detect recombination signals across the dataset [25]. A total of seven detection methods implemented in RDP4 were employed: RDP, GENECONV, 3Seq, Chimaera, SiScan, MaxChi, and BootScan [25]. Recombination events supported by at least three of the seven methods with a *p* < 0.05 were considered credible. Subsequently, we quantified the number of ASFV strains showing significant recombination signals for each gene to assess the distribution and prevalence of recombination events across the genome.

### Selection pressure analyses

To identify the evolutionary pattern of ASFV genes, we selected 23 representative strains based on the phylogenetic tree constructed from the ASFV genome, ensuring that the selected strains covered the main evolutionary branches of the phylogenetic tree for subsequent analysis. We first used the BUSTED method and the MEME method to perform selection pressure analyses on the DATAMONKEY platforms [26, 27]. The BUSTED method was employed to identify gene positive selection. The MEME method was employed to identify site-level positive selection. Given MEME is more permissive approach, we further examined genes that exhibited positive selection signals in both BUSTED and MEME for selective pressures using PAML [28]. We selected the site model (M7 vs. M8) and the free ratio branch model to validate positive selection. Two comparative site models, null model M7 and selection model M8, were used to identify whether a proportion of residues underwent positive selection. The free ratio model (model = 0) that allows the dN/dS values to vary among all branches was used to identify the branches subject to positive selection. Ultimately, genes exhibiting more than five positively selected residues were visualized, with protein structures predicted by AlphaFold 3 [29], and the positively selected sites depicted using PyMOL 3.1 [30].

## Results

### Phylogenetic insights into ASFV evolution

**Figure 1:**
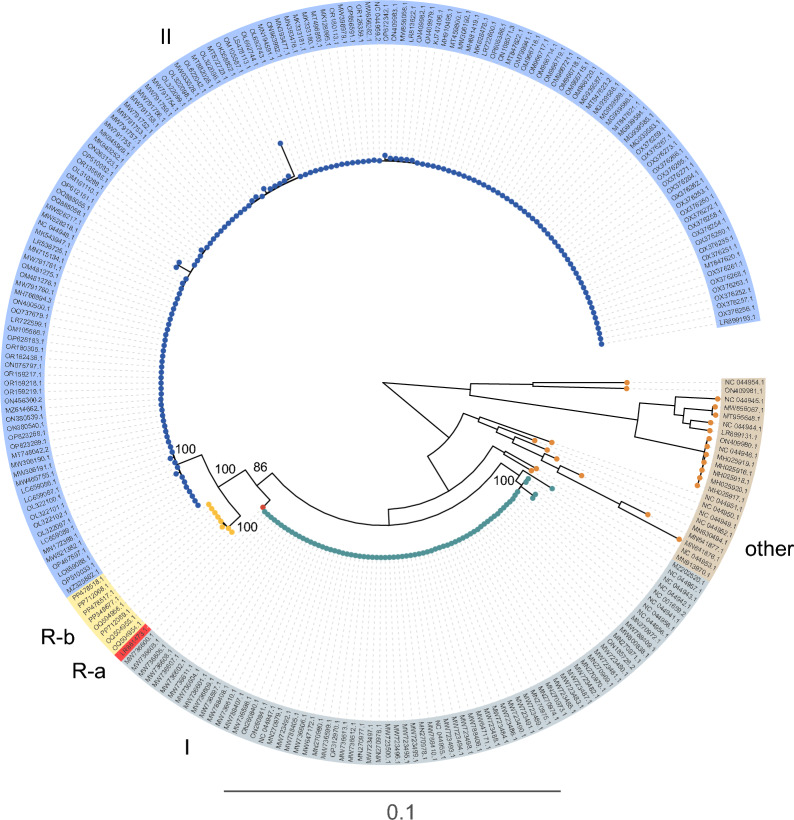
**Phylogenetic tree of ASFV genomes.** The major clades are highlighted in distinct colors.

To unravel the evolutionary trajectory of ASFVs, a comprehensive phylogenetic tree was constructed using 252 complete genomes retrieved from the NCBI database. This analysis revealed that ASFVs can be categorized into three primary clusters, with several minor lineages nested within these groups (Figure 1). Among these, genotypes I and II have long been recognized as the predominant lineages driving the global epidemiology of ASFVs. Intriguingly, recent years have witnessed the emergence of a novel recombinant lineage [12], originating from genetic recombination events between genotypes I and II, which now appears to be establishing itself as a significant evolutionary and epidemiological force.

This recombinant lineage bifurcates into two distinct subgroups, referred to as genotypes R-a and R-b (Figure 1). While genotype R-a is represented by a solitary genome sequence (LR881473.1), genotype R-b comprises a more extensive array of sequences and has rapidly become the dominant recombinant strain (Figure 1). First identified in China in 2022, genotype R-b has since been reported in Vietnam and Russia [31, 32], underscoring its capacity for geographic expansion and its potential for widespread dissemination. Notably, the genomic characteristics and the apparent fitness advantages of genotype R-b suggest that it may pose an increasing threat to global swine production systems.

### Genomic variability and core gene classification of ASFVs

**Figure 2:**
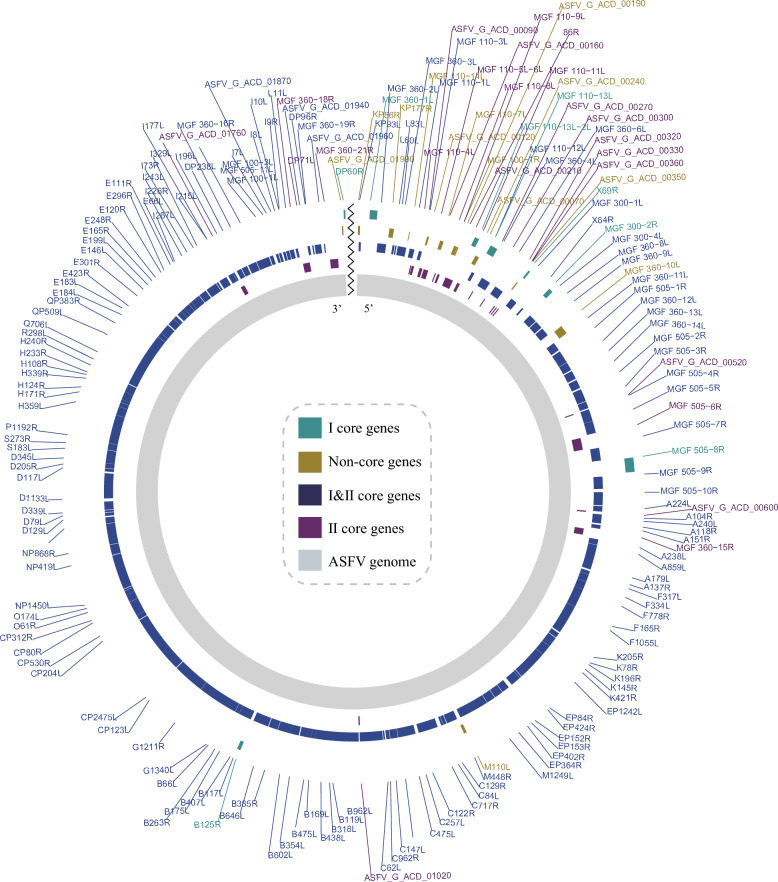
**Classification of ASFV genes.** ASFV genes were systematically classified into four categories based on gene prevalence: I core genes, II core genes, I & II core genes, and non-core genes, each represented by a distinct color.

Given that ASFV is a member of the NCLDV family, characterized by expansive genomes and a notable tolerance for genomic variability, we utilized pan-genome analyses to interrogate the extent and patterns of gene variability across ASFV strains. Based on gene prevalence within ASFV genomes, genes were systematically classified into four distinct categories: I & II core genes (present in over 95% of both genotype I and genotype II strains), I core genes (exceeding 95% presence exclusively in genotype I strains), II core genes (exceeding 95% presence exclusively in genotype II strains), and non-core genes (present in less than 95% of either genotype I or genotype II strains) (Figure 2).

The analysis demonstrated that the majority of ASFV genes are categorized as I & II core genes, underscoring the relatively conserved genomic architecture of ASFV (Figure 2). Eight genes, including MGF 360-1L, MGF 110-13L-2L, MGF 110-13L, X69R, MGF 300-2R, MGF 505-8R, B125R and DP60R, were identified as I core genes (Figure 2), whereas 23 genes, such as MGF 110-4L, 86R and MGF 360-15R, were uniquely classified as II core genes (Figure 2). Meanwhile, 13 genes, including KP86R, KP177R, MGF 110-14L, ASFV_G_ACD_00120, MGF 110-7L, MGF 100-1R, ASFV_G_ACD_00190, ASFV_G_ACD_00070, ASFV_G_ACD_00240, ASFV_G_ACD_00350, MGF 360 − 10L, M110L and ASFV_G_ACD_01990, were designated as non-core genes, reflecting the genomic flexibility and adaptability characteristic of ASFV (Figure 2).

Detailed mapping of the genomic distribution revealed that genes susceptible to loss are predominantly localized in the flanking regions of the ASFV genome, consistent with the well-documented presence of two highly variable regions at the left and right genomic termini (Figure 2). These findings illustrate the equilibrium between the capacity of ASFV for genomic innovation and the retention of a stable gene repertoire, shedding light on its evolutionary adaptability and the potential mechanisms underpinning its persistence and pathogenicity.

### ASFVs inherit genomic framework from the common ancestor

**Figure 3:**
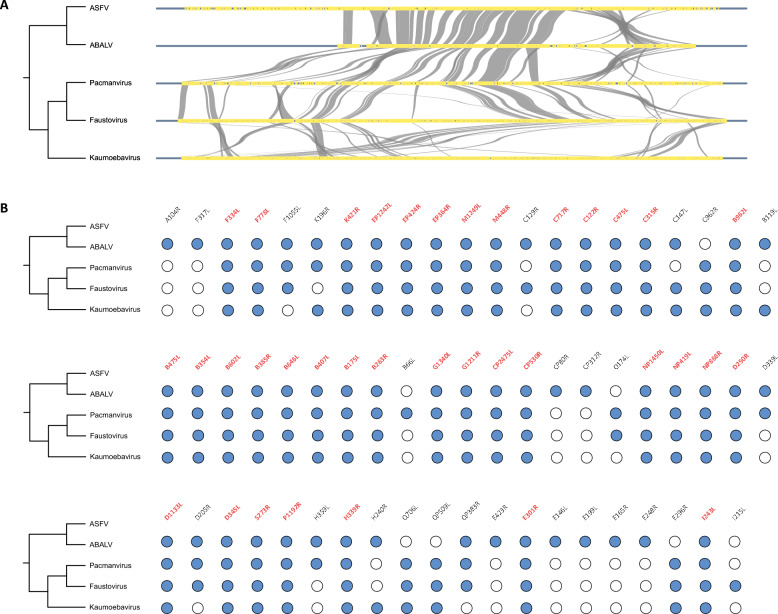
**Synteny analysis of ASFV genes in comparison with four members of the Asfarviridae.** **A** Synteny relationships of shared genes among ASFV, ABALV, Pacmanvirus, Faustovirus, and Kaumoebavirus. **B** Shared genes across five members of the *Asfarviridae*. A blue circle indicates the presence of a gene in a given virus, while a white circle indicates its absence. Genes conserved across all five viruses are highlighted in red.

To further elucidate the evolutionary trajectory of ASFV genes, we conducted gene synteny analyses across five members of the *Asfarviridae* family, including ASFV, Asfarvirus-like virus (ABALV), Pacmanvirus, Faustovirus, and Kaumoebavirus. Our results revealed that approximately 32% (62/194) of ASFV genes are derived from the common ancestor of the *Asfarviridae* family, with these genes predominantly located in the central region of the ASFV genome, forming the core genomic framework (Figure 3A). The conserved gene order between ASFV and ABALV indicates strong gene synteny, while synteny patterns become increasingly divergent among more distantly related members of the family (Figure 3A). Notably, although 62 ASFV genes were found in other *Asfarviridae* viruses, some of these genes have been lost or modified over the course of evolution in certain lineages. Nevertheless, 36 genes are conserved across all five *Asfarviridae* viruses, and all are categorized as type I and II core genes, suggesting that they play essential roles in ASFV infection and pathogenicity (Figure 3B).

The combined results of gene collinearity analysis and previous pan-genome analysis provide a deeper understanding of the origins of ASFV, revealing that the virus inherited a portion of its core gene repertoire from the common ancestor of the *Asfarviridae* family, which forms the fundamental genomic framework of ASFV, with the core genes primarily concentrated in the central region (Figure 3). On this foundation, ASFV acquired a set of virus-specific genes, further shaping its unique genetic identity. Interestingly, genes located at the genomic termini are more susceptible to loss or variation throughout the evolutionary history of ASFV, reflecting the dynamic nature of its genome (Figures 2 and 3).

### Pervasive homologous recombination on ASFV genome

**Figure 4:**
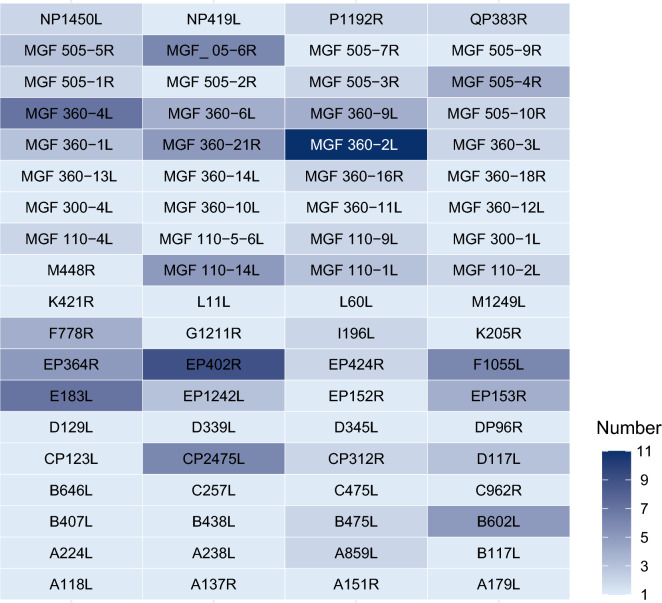
**Recombination analysis of 76 genes with significant recombination signals.** Heatmap illustrating the distribution of recombination signals across ASFV genes. A total of 76 genes showed significant recombination, defined as having at least one ASFV strain identified as recombinant by three or more of the seven detection methods. Color intensity corresponds to the number of ASFV strains exhibiting significant recombination signals for each gene.

To investigate the evolutionary dynamics of ASFV, we conducted recombination analyses across all ASFV genes. Based on the cluster results, 64 representative ASFV strains were selected for these analyses. Among 194 genes, 76 showed significant recombination signals, defined as having at least one ASFV strain identified as recombinant by three or more of the seven detection methods applied (Figure 4). Notably, MGF 360-2L was the most frequently recombined gene, with recombination detected in 11 strains. Other genes with high levels of recombination included EP402R, E183L, MGF 360-4L, CP2475L, F1055L, and MGF 505-6R, each of which showed recombination signals in multiple ASFV strains (Figure 4). Recombination events were not clustered in specific genomic regions but were dispersed throughout the entire ASFV genome (Figures 2 and 4). Genes from all four functional categories exhibited evidence of recombination. Notably, many genes scattered across the ASFV genome displayed strong recombination signals, underscoring the significant role of recombination in ASFV evolution and highlighting its contribution to the emergence of recombinant ASFV strains.

### ASFV genes experienced positive selection

**Figure 5:**
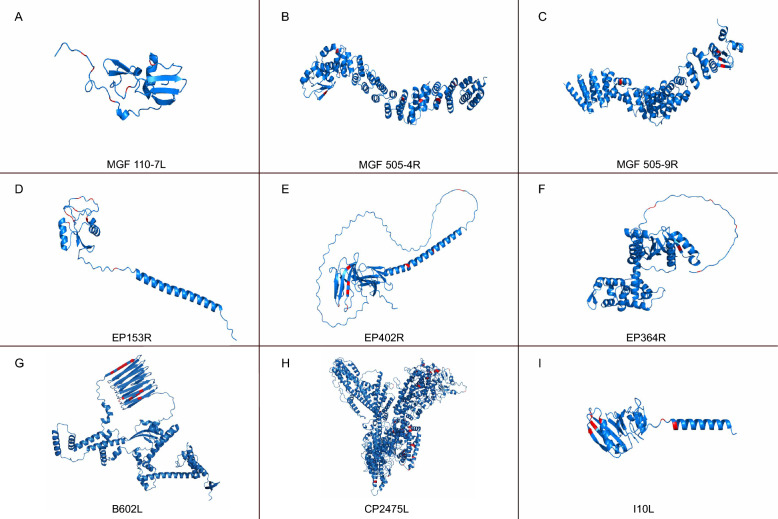
**Positive selection sites identified in ASFV genes.** Positive selection was detected using both MEME and PAML methods, with 9 genes showing more than five positively selected residues. These genes include MGF 110-7L, MGF 505-4R, MGF 505-9R, EP153R, EP402R, EP364R, B602L, CP2475L and I10L. In the predicted tertiary structures of these genes, the positively selected sites are highlighted in red.

**Figure 6:**
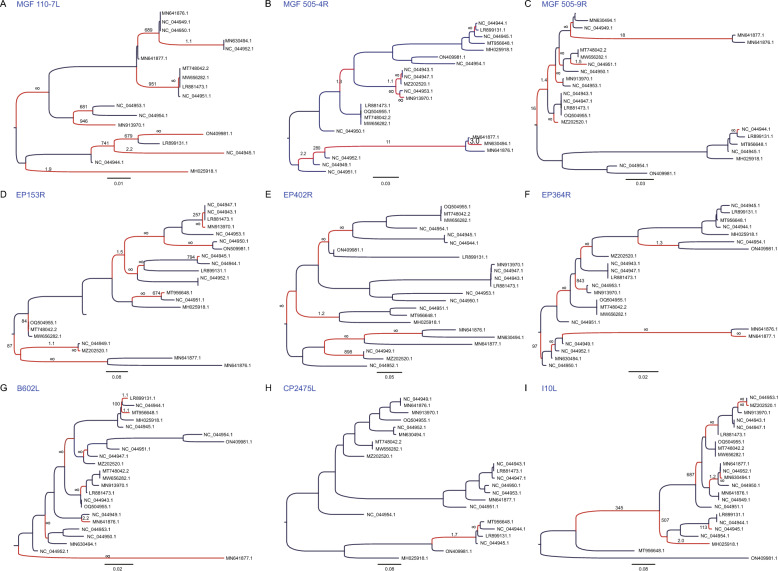
**Positive selection branches in ASFV genes.** Phylogenetic trees were constructed using ASFV genes. Branches under positive selection are highlighted in red and marked with specific ω values.

The evolutionary arms race between hosts and viruses is a major driving force behind viral evolution. To investigate whether ASFV genes have undergone positive selection, we employed the BUSTED method on the DATAMONKEY platform. Our analysis revealed that 55 ASFV genes exhibited strong signals of positive selection (Additional file 1). Additionally, the MEME method was used to examine site-level selection, identifying positively selected residues in 117 genes, with the number of positively selected residues ranging from 1 to 38 per gene (Additional file 1). Given the more permissive approach of MEME, 50 genes that showed positive selection signals in both BUSTED and MEME were further analyzed for selective pressures using PAML. Using the site model (M7 vs. M8), we detected positively selected residues in 29 genes, with nine genes having more than five residues under positive selection, including MGF 110-7L, MGF 505-4R, MGF 505-9R, EP153R, EP402R, EP364R, B602L, CP2475L, and I10L (Figure 5). These genes, predominantly from core classes I and II, except for MGF 110-7L, exhibited strong evolutionary pressures (Figures 2 and 5). Further analysis with the free ratio branch model revealed that all nine genes had branches under positive selection (Figure 6). Our findings underscore the significant role of host-virus evolutionary dynamics in driving the elevated evolution of ASFV genes, despite ASFV proofreading mechanisms as a DNA virus.

## Discussion

The results of this study provide significant new insights into the evolutionary dynamics of ASFV, an economically devastating pathogen responsible for large-scale outbreaks of swine fever globally. Our phylogenetic, genomic, and evolutionary analyses not only deepen our understanding of ASFV evolutionary history but also highlight its remarkable ability to adapt through recombination and selection pressures. These findings have broad implications for the epidemiology, control, and prevention of ASFV, especially in the face of emerging recombinant strains that present unique challenges to global biosecurity [12].

The phylogenetic tree constructed from 252 complete ASFV genomes reveals a complex viral evolution, marked by three primary clusters that align with the established genotypes I and II, and a recently identified recombinant lineage (genotype R) (Figure 1). The discovery of genotype R, which arises from recombination events between genotypes I and II, is particularly striking, as it underscores a significant evolutionary shift in ASFV genomic landscape. Previously, genotypes I and II were regarded as the dominant lineages responsible for ASFV outbreaks, with genotype II being particularly prominent in Europe and Asia since the disease resurfaced in these regions in the early 2000s [33, 34]. However, the emergence of genotype R signals the rapid evolution of ASFV in response to shifting epidemiological pressures. Since its first detection in China in 2022, genotype R-b has rapidly spread to Vietnam and Russia, reinforcing the potential for geographic expansion of this recombinant strain [12, 31, 32]. The global spread of genotype R-b further emphasizes the adaptability of ASFV and suggests it may become a major force in the future epidemiology (Figure 1). The recombination between genotypes I and II may have conferred unique advantages to R-b in terms of transmission dynamics, virulence, or immune evasion, making it a priority for continued monitoring and surveillance in affected regions.

The rapid emergence and spread of recombinant ASFV strains such as genotype R-b highlight the complex role of genetic recombination in ASFV evolution. Unlike many viruses, ASFV is a large, double-stranded DNA virus, and its recombination potential is significant, given its large genome and propensity for genetic diversity. Compared with previous studies that focused on genome-level recombination patterns, our study conducted recombination analysis at the gene level, enabling a more fine-grained understanding of how specific coding regions are affected by recombination [35]. This gene-centric approach allowed us to pinpoint functionally relevant genes—such as EP402R, E183L, and members of the MGF 360/505 families—as recurrent targets of recombination events. Recombination events in ASFV are not clustered in any specific genomic region but are spread throughout the genome, suggesting a high degree of genomic plasticity that facilitates viral adaptation. The significance of recombination in ASFV evolution cannot be overstated (Figure 4). Recombination allows ASFV to rapidly shuffle genetic material, facilitating the emergence of new virus variants that may have enhanced fitness. This is particularly important for ASFV, given its ability to infect wide host populations across different geographic regions. The emergence of genotype R-b, therefore, represents an example of how recombination can generate new viral variants with enhanced transmission potential or altered pathogenic characteristics, posing challenges to existing control strategies and vaccine development (Figures 1 and 4).

Beyond recombination, another key feature of ASFV evolution is its genomic flexibility. The pan-genome analysis reveals that ASFV core genome is highly conserved, with a substantial proportion of genes shared between genotypes I and II. These core genes, which account for over 95% of both genotypes, play essential roles in the viral lifecycle, including replication, immune evasion, and host cell manipulation [36, 37]. However, the presence of genotype-specific core genes and non-core genes, which are present in less than 95% of the viral strains, underscores the adaptability of ASFV (Figure 2). Non-core genes may allow ASFV to adapt to different host species, geographic regions, or environmental conditions, contributing to the ability of ASFV to persist and spread globally. This genomic flexibility is further highlighted by the presence of variable regions at the genomic termini, which are more prone to gene loss and variation [8]. These flanking regions may harbor genes that are dispensable or subject to modification as the virus adapts to new ecological niches or evades host immune responses (Figure 2). In this regard, the balance between maintaining a conserved core genome and allowing for genomic variability in non-essential regions is crucial for the long-term persistence and pathogenicity of ASFV (Figure 3).

The results of the gene synteny analysis further clarify the evolutionary trajectory of ASFV, particularly its relationship with other members of the *Asfarviridae* family (Figure 3). This analysis revealed that approximately 32% of ASFV genes are conserved across the family, with a core set of genes inherited from the common ancestor of the *Asfarviridae* family. These genes, primarily located in the central region of the genome, form the backbone of ASFV genomic architecture and are likely involved in fundamental processes such as viral replication and host interaction. However, despite this conservation, ASFV has undergone substantial genomic diversification, acquiring virus-specific genes that further shape its unique genetic identity (Figures 2 and 3). The divergence of ASFV genomic structure from its relatives, such as Pacmanvirus and Faustovirus, indicates that ASFV has adapted to a specialized niche as a pathogen of swine, which may involve unique interactions with the host immune system or the exploitation of specific host cell machinery.

This model of viral evolution, which combines inheritance from an ancestral lineage with the continuous acquisition of novel genetic material through recombination and other mechanisms, underscores the remarkable adaptability of ASFV. It also emphasizes the ability of ASFV to maintain genomic stability in essential regions while diversifying in other parts of the genome, a balance that is key to its success as a highly adaptable pathogen. As ASFV continues to spread and evolve, its capacity for both genomic stability and variability will remain central to its ability to cause persistent and widespread outbreaks, posing ongoing challenges for control and eradication strategies.

While recombination provides a mechanism for generating genetic diversity, selection pressures, particularly those imposed by host immune responses, play a crucial role in shaping ASFV evolution. The evolutionary arms race between viruses and their hosts is a central theme in viral evolution [38], and ASFV is no exception. In this study, we identified 9 genes under strong positive selection, indicating that these genes are subject to intense evolutionary pressure to adapt to host defenses (Additional file 1). The process of positive selection favors mutations that enhance viral survival and fitness, particularly those that help the virus evade immune recognition, facilitate replication, or increase transmission.

Interestingly, despite being a DNA virus with relatively lower mutation rates compared to RNA viruses [19], ASFV exhibits a remarkable capacity for evolutionary adaptation. The constant evolutionary arms race, in which the virus is perpetually pressured to evolve immune evasion strategies while the host immune system adapts to recognize and counteract the virus, serves as a major driving force behind the rapid evolution of ASFV. The identification of specific positively selected residues within these immune-related genes, such as EP153R, EP402R, EP364R, B602L and I10L (Figure 5 and 6), highlights the antagonistic interactions between the ASFV and the host, providing valuable insights into potential critical amino acid residues for further investigation into ASFV protein functions [39–42]. It is worth emphasizing that other genes, such as MGF 110-7L, MGF 505-4R, MGF 505-9R and CP2475L (Figure 5 and 6), also manifest pronounced positive selection signatures, suggesting they could play a pivotal role in the initial antagonistic interface between ASFV and the host, although this warrants further corroboration.

In conclusion, this study provides a detailed examination of the evolutionary trajectory of ASFV, shedding light on the complex processes that drive its adaptation and persistence. The findings underscore the remarkable genomic flexibility of ASFV, its capacity for homologous recombination, and the selective pressures that shape its evolution. As ASFV continues to spread globally and adapt to hosts and environments, continued research into its genomic structure and evolutionary dynamics will be essential for developing effective strategies to combat this devastating disease.

## Supplementary Information

**Additional file 1. Results of selection pressure analyses of ASFV genes.**

## Data Availability

All data generated or analysed during this study are included in this published article and its supplementary information files.
