# Supplementary material for: Homologous recombination and evolutionary arms race drive the adaptive evolution of African swine fever virus
Source: Vet Res. 2025 Jul 8;56:142. doi: 10.1186/s13567-025-01573-1 (PMC12235792; doi:10.1186/s13567-025-01573-1)
Supplement: Supplementary file 1 — Additional file 1. Results of selection pressure analyses of ASFV genes. [file 13567_2025_1573_MOESM1_ESM.docx]

**Additional file 1 Results of selection pressure analyses of ASFV genes.**

| Gene | BUSTED | MEME | PAML |
| --- | --- | --- | --- |
| 86R | p=0.1392 | 0 | 0 |
| A104R | p=0.5000 | 0 | 0 |
| A118R | p=0.5000 | 5 | 0 |
| A137R | p=0.3247 | 1 | 0 |
| A151R | p=0.2323 | 3 | 0 |
| A179L | p=0.5000 | 0 | 0 |
| A224L | p=0.5000 | 1 | 0 |
| A238L | p=0.09398 | 1 | 0 |
| A240L | p=0.1061 | 2 | 0 |
| A859L | p=0.5000 | 1 | 0 |
| ASFV_G_ACD_00070 | p=0.002250 | 0 | 0 |
| ASFV_G_ACD_00160 | p=0.1458 | 0 | 0 |
| ASFV_G_ACD_00360 | p=0.3117 | 0 | 0 |
| ASFV_G_ACD_00520 | p=0.4160 | 1 | 0 |
| ASFV_G_ACD_00600 | p=0.5000 | 0 | 0 |
| ASFV_G_ACD_01020 | p=0.5000 | 0 | 0 |
| ASFV_G_ACD_01760 | p=0.01821 | 6 | 1 |
| ASFV_G_ACD_01870 | p=0.4983 | 0 | 0 |
| ASFV_G_ACD_01940 | p=0.1225 | 0 | 0 |
| ASFV_G_ACD_01990 | p=0.07651 | 1 | 0 |
| B117L | p=0.003411 | 2 | 4 |
| B119L | p=0.5000 | 0 | 0 |
| B125R | p=0.5000 | 0 | 0 |
| B169L | p=0.3615 | 0 | 0 |
| B175L | p=0.5000 | 0 | 0 |
| B263R | p=0.5000 | 0 | 0 |
| B318L | p=0.5000 | 0 | 0 |
| B354L | p=0.4273 | 0 | 0 |
| B385R | p=0.000008667 | 3 | 0 |
| B407L | p=0.3078 | 1 | 0 |
| B438L | p=0.2745 | 1 | 0 |
| B475L | p=0.009147 | 4 | 1 |
| B602L | p=1.127e-14 | 23 | 11 |
| B646L | p=0.5000 | 0 | 0 |
| B66L | p=0.5000 | 0 | 0 |
| B962L | p=0.4971 | 1 | 0 |
| C122R | p=0.0003602 | 1 | 0 |
| C129R | p=0.5000 | 0 | 0 |
| C147L | p=0.3833 | 1 | 0 |
| C257L | p=0.5000 | 0 | 0 |
| C315R | p=0.5000 | 0 | 0 |
| C475L | p=0.5000 | 1 | 0 |
| C62L | p=0.5000 | 0 | 0 |
| C717R | p=0.01532 | 2 | 0 |
| C84L | p=0.04476 | 0 | 0 |
| C962R | p=0.5000 | 2 | 0 |
| CP123L | p=0.01417 | 1 | 0 |
| CP204L | p=0.5000 | 3 | 0 |
| CP2475L | p=1.038e-7 | 38 | 40 |
| CP312R | p=0.5000 | 2 | 0 |
| CP530R | p=0.2376 | 1 | 0 |
| CP80R | p=0.5000 | 0 | 0 |
| D1133L | p=0.3140 | 3 | 0 |
| D117L | p=0.5000 | 1 | 0 |
| D129L | p=0.0001432 | 2 | 1 |
| D205R | p=0.03007 | 1 | 0 |
| D250R | p=0.5000 | 0 | 0 |
| D339L | p=0.5000 | 0 | 0 |
| D345L | p=0.5000 | 0 | 0 |
| D79L | p=0.5000 | 0 | 0 |
| DP238L | p=0.4954 | 3 | 0 |
| DP60R | p=0.09583 | 1 | 0 |
| DP71L | p=0.006817 | 1 | 0 |
| DP96R | p=0.06022 | 3 | 0 |
| E111R | p=0.5000 | 0 | 0 |
| E120R | p=0.5000 | 1 | 0 |
| E146L | p=0.4710 | 1 | 0 |
| E165R | p=0.5000 | 0 | 0 |
| E183L | p=0.002968 | 4 | 1 |
| E184L | p=0.02244 | 1 | 0 |
| E199L | p=0.5000 | 1 | 0 |
| E248R | p=0.5000 | 1 | 0 |
| E296R | p=0.5000 | 0 | 0 |
| E301R | p=0.5000 | 0 | 0 |
| E423R | p=0.2355 | 2 | 0 |
| E66L | p=2.325e-7 | 4 | 2 |
| EP1242L | p=0.5000 | 0 | 0 |
| EP152R | p=0.5000 | 0 | 0 |
| EP153R | p=0.0003632 | 14 | 9 |
| EP364R | p=0.003008 | 11 | 6 |
| EP402R | p=5.767e-13 | 19 | 9 |
| EP424R | p=0.5000 | 0 | 0 |
| EP84R | p=0.5000 | 0 | 0 |
| F1055L | p=3.062e-7 | 13 | 1 |
| F165R | p=0.1407 | 1 | 0 |
| F317L | p=0.5000 | 1 | 0 |
| F334L | p=0.5000 | 0 | 0 |
| F778R | p=0.5000 | 6 | 0 |
| G1211R | p=0.000006190 | 7 | 1 |
| G1340L | p=0.5000 | 3 | 0 |
| H108R | p=0.2342 | 1 | 0 |
| H124R | p=0.5000 | 0 | 0 |
| H171R | p=0.5000 | 0 | 0 |
| H233R | p=0.009287 | 4 | 0 |
| H240R | p=0.08206 | 4 | 0 |
| H339R | p=0.5000 | 1 | 0 |
| H359L | p=0.5000 | 0 | 0 |
| I10L | p=0.0002721 | 10 | 8 |
| I177L | p=0.01080 | 5 | 1 |
| I196L | p=0.5000 | 2 | 0 |
| I215L | p=0.02390 | 1 | 4 |
| I226R | p=0.1028 | 2 | 0 |
| I243L | p=0.0008495 | 3 | 0 |
| I267L | p=0.5000 | 0 | 0 |
| I329L | p=0.5000 | 1 | 0 |
| I73R | p=0.3214 | 1 | 0 |
| I7L | p=0.3970 | 0 | 0 |
| I8L | p=0.001727 | 1 | 0 |
| I9R | p=0.02138 | 0 | 0 |
| K145R | p=0.5000 | 0 | 0 |
| K196R | p=0.5000 | 0 | 0 |
| K205R | p=0.4922 | 1 | 0 |
| K421R | p=0.2584 | 1 | 0 |
| K78R | p=0.4945 | 0 | 0 |
| KP177R | p=0.00002878 | 0 | 0 |
| KP86R | p=0.5000 | 0 | 0 |
| KP93L | p=0.3610 | 0 | 0 |
| L11L | p=0.3557 | 0 | 0 |
| L60L | p=0.06315 | 1 | 0 |
| L83L | p=0.003523 | 1 | 0 |
| M110L | p=0.1096 | 0 | 0 |
| M1249L | p=0.5000 | 1 | 0 |
| M448R | p=0.5000 | 1 | 0 |
| MGF 100-1L | p=0.0004059 | 2 | 0 |
| MGF 100-1R | p=0.5000 | 0 | 0 |
| MGF 100-3L | p=0.006005 | 3 | 1 |
| MGF 110-11L | p=7.819e-7 | 5 | 0 |
| MGF 110-12 | p=0.0009704 | 1 | 0 |
| MGF 110-14L | p=4.940e-15 | 9 | 0 |
| MGF 110-1L | p=0.0002126 | 10 | 0 |
| MGF 110-2L | p=0.003934 | 1 | 0 |
| MGF 110-3L | p=0.001983 | 4 | 1 |
| MGF 110-4L | p=0.1621 | 2 | 0 |
| MGF 110-5-6L | p=0.01436 | 3 | 1 |
| MGF 110-7L | p=0.0006491 | 5 | 5 |
| MGF 110-8L | p=0.02154 | 0 | 0 |
| MGF 110-9L | p=0.01350 | 3 | 1 |
| MGF 300-1L | p=0.5000 | 5 | 0 |
| MGF 300-2R | p=0.5000 | 2 | 0 |
| MGF 300-4L | p=0.5000 | 3 | 0 |
| MGF 360-10L | p=0.1289 | 3 | 0 |
| MGF 360-11L | p=0.5000 | 0 | 0 |
| MGF 360-12L | p=0.04418 | 1 | 1 |
| MGF 360-13L | p=0.4782 | 4 | 0 |
| MGF 360-14L | p=0.001040 | 3 | 1 |
| MGF 360-15R | p=0.5000 | 3 | 0 |
| MGF 360-16R | p=0.001020 | 3 | 0 |
| MGF 360-18R | p=0.2175 | 2 | 0 |
| MGF 360-19R | p=0.5000 | 1 | 0 |
| MGF 360-1L | p=0.2277 | 0 | 0 |
| MGF 360-21R | p=0.09581 | 7 | 0 |
| MGF 360-2L | p=0.1073 | 4 | 0 |
| MGF 360-3L | p=0.5000 | 3 | 0 |
| MGF 360-4L | p=0.003033 | 6 | 2 |
| MGF 360-6L | p=0.02816 | 4 | 1 |
| MGF 360-8L | p=0.2894 | 4 | 0 |
| MGF 360-9L | p=0.1644 | 4 | 0 |
| MGF 505-10R | p=0.1322 | 7 | 0 |
| MGF 505-11L | p=0.5000 | 0 | 0 |
| MGF 505-1R | p=0.04994 | 7 | 3 |
| MGF 505-2R | p=0.5000 | 3 | 0 |
| MGF 505-3R | p=0.01954 | 7 | 0 |
| MGF 505-4R | p=0.00001173 | 14 | 8 |
| MGF 505-5R | p=0.1444 | 7 | 0 |
| MGF 505-6R | p=0.1969 | 10 | 0 |
| MGF 505-7R | p=0.5000 | 3 | 0 |
| MGF 505-9R | p=0.03881 | 12 | 5 |
| NP1450L | p=0.002016 | 1 | 0 |
| NP419L | p=0.5000 | 0 | 0 |
| NP868R | p=0.5000 | 1 | 0 |
| O174L | p=0.2443 | 1 | 0 |
| O61R | p=0.5000 | 0 | 0 |
| P1192R | p=0.0007038 | 2 | 0 |
| Q706L | p=0.4519 | 0 | 0 |
| QP383R | p=0.0007796 | 2 | 1 |
| QP509L | p=0.5000 | 0 | 0 |
| R298L | p=0.5000 | 2 | 0 |
| S183L | p=0.5000 | 0 | 0 |
| S273R | p=0.5000 | 0 | 0 |
| X64R | p=0.08927 | 0 | 0 |
| X69R | p=0.1334 | 4 | 0 |
